# Sabirnetug shows superior selectivity for Aβ oligomers over monomer compared to recombinant lecanemab and aducanumab

**DOI:** 10.1002/alz70859_103130

**Published:** 2025-12-25

**Authors:** Erika N Cline, Elizabeth Johnson, Martin Kleinschmidt, Mathias Schenk, Thomas Lavoie, Jens‐Ulrich Rahfeld, Stephan Schilling, Jasna Jerecic

**Affiliations:** ^1^ Acumen Pharmaceuticals, Newton, MA USA; ^2^ Fraunhofer Institute for Cell Therapy and Immunology, Halle Germany

## Abstract

**Background:**

Sabirnetug (ACU193) is a monoclonal antibody designed to selectively target toxic soluble amyloid β oligomers (AβOs). Its efficacy in mild cognitive impairment (MCI) and early Alzheimer’s disease (AD) is currently being investigated in the ALTITUDE‐AD phase 2 study (NCT06335173). Therapeutic targeting of AβOs in MCI/AD is challenged by the significantly higher abundance of the less toxic Aβ monomers in MCI/AD tissues and biofluids, e.g., ∼7,000‐fold for Aβ_1‐40_/AβOs in CSF. Therefore, the therapeutic efficacy of AβO‐targeting antibodies is enhanced by high selectivity for AβOs over monomeric Aβ. Here, we compare the binding affinities to AβOs and Aβ monomer of sabirnetug with other Aβ‐targeting antibodies lecanemab, aducanumab, and murine donanemab.

**Method:**

Human lecanemab and aducanumab and murine donanemab were recombinantly produced from published sequences. Binding kinetics of these antibodies or sabirnetug to AβOs (ADDLs or Aβ_1‐42_ stabilized oligomers from GBS Leiden) or monomeric Aβ proteoforms (Aβ_1‐28_, Aβ_1‐40_, Aβ_1‐42_) was determined by surface plasmon resonance (SPR). Briefly, each Aβ test species was captured by anti‐Aβ antibody 6E10 conjugated to the SPR chip and then the test antibody was titrated across the chip.

**Result:**

Sabirnetug showed the highest binding affinities to both AβO preparations with a correspondingly low K_D_ = 0.596 nM for Aβ_1‐42_ stabilized oligomers and 0.319 nM for ADDLs. Lecanemab had the next highest binding affinities at 0.972 nM and 0.762 nM, respectively. Sabirnetug showed the lowest binding affinities to monomeric Aβ_1‐40_ (sabirnetug >5.22 µM; lecanemab, 215.7 nM) and a comparable affinity to monomeric Aβ_1‐28_ as lecanemab (sabirnetug, 4.98 µM; lecanemab, 9.38 µM). Measurement of monomeric Aβ_1‐42_ binding was confounded by oligomerization within the experimental time frame. Sabirnetug had the highest selectivity for AβOs over monomeric Aβ_1‐40_, e.g., 8,750‐fold selectivity for Aβ_1‐42_ stabilized oligomers over Aβ_1‐40_ compared to 222‐fold selectivity of lecanemab, and comparable selectivity with lecanemab for AβOs over monomeric Aβ_1‐28_. Aducanumab and murine donanemab were less selective for AβOs over both monomeric Aβ proteoforms.

**Conclusion:**

Compared to the recombinant Aβ monoclonal antibody therapeutics tested, sabirnetug showed the highest selectivity for AβOs over monomeric Aβ. Sabirnetug’s observed high level of selectivity makes it well positioned to target AβOs in MCI/AD tissues and biofluids.

